# Circadian Gene Variants in Diseases

**DOI:** 10.3390/genes14091703

**Published:** 2023-08-27

**Authors:** Paula Gršković, Petra Korać

**Affiliations:** Division of Molecular Biology, Department of Biology, Faculty of Science, University of Zagreb, 10 000 Zagreb, Croatia; paula.grskovic@biol.pmf.unizg.hr

**Keywords:** circadian gene variants, tumors, metabolic diseases, cardiovascular diseases, sleep disorders

## Abstract

The circadian rhythm is a self-sustaining 24 h cycle that regulates physiological processes within the body, including cycles of alertness and sleepiness. Cells have their own intrinsic clock, which consists of several proteins that regulate the circadian rhythm of each individual cell. The core of the molecular clock in human cells consists of four main circadian proteins that work in pairs. The CLOCK-BMAL1 heterodimer and the PER-CRY heterodimer each regulate the other pair’s expression, forming a negative feedback loop. Several other proteins are involved in regulating the expression of the main circadian genes, and can therefore also influence the circadian rhythm of cells. This review focuses on the existing knowledge regarding circadian gene variants in both the main and secondary circadian genes, and their association with various diseases, such as tumors, metabolic diseases, cardiovascular diseases, and sleep disorders.

## 1. Introduction

The circadian system is a complex multioscillatory temporal network in which an ensemble of coupled neurons comprising the principal circadian pacemaker in the suprachiasmatic nucleus (SCN) of the hypothalamus is entrained to the daily light/dark cycle, and subsequently transmits synchronizing signals to peripheral clocks in all cells of the body. Peripheral clocks are a part of a cell’s physiology that regulates the cell’s circadian cycle according to the signals coming from the main clock in the SCN [1]. The importance of well-tuned mechanisms regulating circadian rhythm for health and survival is implied by their presence in a wide variety of both prokaryotic and eukaryotic species [2]. Complex organisms, including humans, have developed a central clock, consisting of several proteins, that regulates the body’s response under the influence of environmental conditions (so-called “zeitgebers”—time givers) such as light and temperature. In humans and other mammals, this center is located in the SCN of the hypothalamus, from which signals are dispensed to peripheral tissues in order to synchronize the circadian rhythm of the peripheral clocks in each individual cell with that of the main clock. While cells separated from the main clock still possess autonomic regulation of circadian cycle in vitro [3], signals from a wild-type SCN have been shown to override the lack of a functioning intrinsic clock caused by mutations in circadian genes in cells [4,5], indicating a complex network of interactions between the main clock and peripheral clocks through various pathways [1].

**Figure 1:**
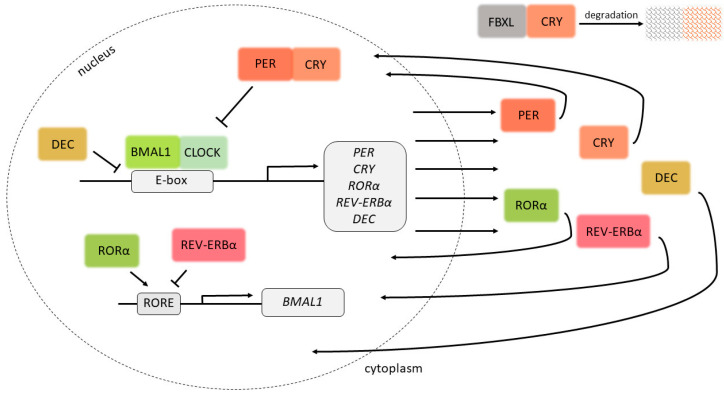
Circadian clock proteins and their negative feedback loop. By binding to E-boxes in DNA, the BMAL1-CLOCK heterodimer induces the expression of target genes, including *PER* and *CRY*, the protein products of which inhibit the expression of *BMAL1* and *CLOCK*. The lack of BMAL1 and CLOCK inhibits the expression of CRY and PER, causing the cycle to start anew. DEC1 and DEC2 compete with the BMAL1-CLOCK heterodimer for the binding of E-boxes, and are therefore involved in the regulation of expression of the target genes. By binding ROR response elements (ROREs), RORα induces the expression of BMAL1, while REV-ERBα inhibits the expression of BMAL1. The activity of CRY1 is also regulated by its interaction with FBXL proteins, which regulate its degradation.

The core of the molecular clock in cells consists of four main proteins, each of which regulates the expression of the others, with these expressions fluctuating over the course of a 24 h period, as well as the expression of other downstream genes [6]. The first of the four to be described was the *period* gene (*per*) in *Drosophila melanogaster* in 1971 [7]. Its role in the regulation of the molecular clock was confirmed by restoring circadian rhythm in mutant flies by introducing a wild-type *per* allele [8,9]. Three ortholog genes, *Per1*, *Per2* and *Per3*, have been described in mammals, and their mRNA and protein levels have also been shown to vary over the sleeping–wakefulness period [10,11,12,13]. The expressions of *PER1*, *PER2* and *PER3* are regulated by a heterodimeric protein consisting of the transcription factors CLOCK (*circadian locomotor out-put cycles kaput*) and BMAL1 (*Brain and Muscle ARNT-Like 1*, now denoted *ARNT-Like* or *Arntl*), whose expressions are in turn regulated by PER and CRY in a negative feedback loop (Figure 1).

*Clock* was first described in mice as a gene encoding a regulator of the intrinsic circadian period and the persistence of circadian rhythmicity in conditions of constant darkness [14]. Its expression varies between different tissues and its sequence is largely conserved among species [15]. CLOCK contains a PAS dimerization domain (named for the *per*, *ARNT* and *sim* genes it was initially associated with), through which it binds to BMAL1 in order to form a heterodimer that binds to DNA through the basic helix–loop–helix DNA-binding domain [16]. The localization of CLOCK in the nucleus is dependent on the expression of BMAL1 and its binding to CLOCK [17]. The final component of circadian clocks corresponds to the cryptochrome genes, *Cry1* and *Cry2*, which code for two blue-light photoreceptors. These proteins bind to PER proteins in order to form heterodimers that bind to DNA and regulate gene expression, including the expression of *CLOCK* and *BMAL1* [18,19].

Aside from this feedback loop, there are several additional pathways that regulate the expression of circadian genes. ROR (*Retinoid-related orphan receptors*) transcription factors increase the expression of *BMAL1*, *CRY1* and *REV-ERBα* through interactions with ROR response elements (ROREs) [20,21]. REV-ERBα, whose expression is inhibited by PER and CRYs, regulates the expression of BMAL1 [22], while NAPS2 can partially compensate for the lack of functional CLOCK protein [23]. FBXL3 and FBXL21 regulate the degradation of CRY1 [24,25]. Dec1 and Dec2 inhibit the expression of Per1 by competing with Clock for the binding of Bmal1 or by competing with Clock-Bmal1 heterodimers for the binding of E-boxes, DNA sequences that both Clock-Bmal1 heterodimers and Dec1 and Dec2 are able to bind to [26]. TIMELESS (TIM) regulates the expression–suppression activation of PER1 by regulating its entry to the nucleus [27]. Circadian proteins are also regulated on a post-translational level by reversible phosphorylation, involving several kinases/phosphatases, which affect the stability, activity and localization of these proteins [28].

Pathological changes associated with circadian genes have been described in various types of disease, including tumors [29,30,31], metabolic diseases [32], cardiac diseases [33,34], sleep disorders [35], psychiatric diseases [36] and neurodegenerative diseases [37]. This review focuses on the existing knowledge regarding circadian gene SNPs (single-nucleotide polymorphisms) and their association with these diseases. The investigated SNPs in these studies were selected on the basis of their position within the gene and their predicted function, identified according to several different databases containing data from genome-wide association studies (GWAS), such as the SNPinfo Web Server [38] and the NCBI dbSNP database [39], or by using the Tagger algorithm, implemented in the Haploview interface of HapMap’s genome browser [40,41]. The circadian gene variations in patient cohorts in the studies covered in this review were commonly determined from blood or saliva samples. Alleles whose frequency did not fit into the Hardy–Weinberg equilibrium were excluded from these studies, as it was impossible to estimate their influence on a particular disease.

## 2. Circadian Gene Variants in Tumors

### 2.1. Associations between Circadian Gene Variants and Increased Risk for Tumor Development

Associations between variants of different circadian genes and increased risk for tumor development have been observed in various tumor types (Table 1). The significance of the associations varied depending on whether they were evaluated using a dominant genetic model (DM), a recessive genetic model (RM) or an additive model (AM). DM involve comparison between homozygotes for major alleles (MM) and the combined carriers of minor alleles (Mm + mm), RMs involve comparison between combined carriers of major alleles (MM + Mm) and homozygotes for minor alleles (mm), while AMs involve comparisons among all three genotypes (MM vs. Mm vs. mm) [42].

### 2.2. Associations between Circadian Gene Variants and Increased Risk for Development of Different Tumor Subtypes

The risk for breast cancer (BC) development associated with circadian gene variants was also found to depend on estrogen/progesterone status, as well as on pre-/postmenopausal status. The AA genotype in the *PER1* rs2735611 SNP was associated with higher BC risk based on an RM in Polish women of Caucasian origins when observing the whole cohort as well as when estrogen/progesterone-positive and negative BC were observed separately. In the same cohort, the T allele in the *PER2* rs934945 SNP was generally associated with higher risk of development of breast tumors, as well as estrogen-/progesterone-positive breast tumors based on a DM. Additionally, the risk of development of estrogen-negative tumors was associated with the GG genotype of the *CRY2* rs10838524 SNP based on an RM [43]. In a predominantly Caucasian cohort, the CC genotype in the *CRY2* rs1401417 variant was associated with increased risk of development of BC in postmenopausal patients compared to the GG genotype [55]. In the same study, the *CRY2* rs1401417 C allele, as well as three other SNPs in the same gene (rs11038689 G allele (DM), rs11605924 CC genotype (RM) and rs7123390 A allele (DM)), was found to be significantly associated with risk of estrogen-/progesterone-negative tumors [55]. The *CLOCK* rs3805151 T allele was associated with an increased risk of BC in postmenopausal women based on a DM in Chinese populations [45]. Zhu et al. showed that a *PER3* variant containing five variable number tandem repeats (VNTR; rs57875989) was associated with higher risk of developing BC compared to a *PER3* variant with four VNTRs in young Caucasian women [44] (Table 1), but this association was not observed in other studies, which included Chinese [45], Indian [56] and multiple European cohorts [57]. In a predominantly Caucasian cohort, the heterozygous genotype in the *NPAS2* rs2305160 SNP was associated with greater BC risk in both pre- and postmenopausal women, as well as in the whole cohort [48]. The same locus was also associated with higher risk of BC in women of various ethnicities exposed to rotating shift work for longer periods of time [58].

Aside from the higher risk of developing BC, as described above, the C allele in the *CRY2* rs1401417 SNP was also associated with higher risk of developing prostate cancer (PC) compared to the GG genotype based on a DM in a Chinese population. The risk of developing PC was even greater in patients with the C allele, who also displayed greater insulin resistance (IR) compared to patients with the GG genotype and lower IR [50]. A higher risk for the progression of both localized and advanced PC, as well as poorer survival, was also found to be associated with the T allele in the rs6542993 SNP of the *NPAS2* gene compared to the AA genotype in a predominantly Taiwanese population [59]. Zhu et al. found four variants associated with risk of developing PC (Table 1), but also an additional four SNPs that were associated with the risk of a more aggressive tumor (*CLOCK* 11133373, *NPAS2* rs895521, *PER1* rs885747 and *PER1* rs2289591 variants) and eight SNPs that were significantly associated with risk of less aggressive PC (*PER3* rs1012477, *CRY2* rs2292912, *BMAL1* rs7950226, *NPAS2* rs17024926 and rs1369481, *CSNK1E* rs1534891, *CRY1* rs12315175 and *PER2* rs7602358) in Caucasian men [49]. In contrast, none of the 872 analyzed SNPs in the study by Wendeu-Foyet et al. were significantly associated with risk of developing PC in a French Caucasian cohort [60]. Similarly, Markt et al. did not observe a consistent association between any of the 96 SNPs analyzed and fatal PC across three studied cohorts of European ancestry [61].

Three *CRY2* SNPs, rs11038689, rs7123390 and rs1401417, were found to be associated with risk of developing non-Hodgkin lymphoma (NHL) in a predominantly Caucasian cohort consisting only of female patients. Aside from the whole NHL cohort, the same SNPs were also associated with risk for tumor development in B-cell lymphomas and follicular lymphoma (FL) subgroups (Table 1). Two of those SNPs, rs7123390 (genotype AA) and rs1401417 (genotype CC), were also significantly associated with B-cell chronic lymphocytic leukemia/prolymphocytic leukemia/small lymphocytic lymphoma (CLL/SLL). The CC genotype of the *CRY2* rs1401417 SNP was also associated with the risk of developing diffuse large B-cell lymphoma (DLBCL) and T-cell lymphoma, but no *CRY2* SNPs were significantly associated with risk of developing marginal zone B-cell lymphoma (MZBL) [54].

### 2.3. Associations between Circadian Gene Variants and Decreased Risk for Tumor Development

The *CRY1* rs1056560 GT genotype was associated with a decreased risk for BC in Chinese populations [45]. In the same cohort, premenopausal carriers of the CC genotype in the *CRY2* rs1401417 SNP had a significantly decreased risk of developing BC compared to carriers of the GG genotype, but this association was not observed in postmenopausal women. Decreased risk for BC was also observed for the CC genotype in the *CRY2* rs1401417 SNP in estrogen-positive cases compared to in estrogen-negative cases [45]. Patients carrying the T allele in the *BMAL1* rs2279287 SNP had reduced risk of developing BC [43], and the A allele in the *BMAL1* rs3816358 SNP was also associated with reduced risk for BC [62]. Fu et al. observed that the C allele in the *TIMELESS* rs7302060 SNP was associated with reduced BC risk, as well as that the GG genotype in the *TIMELESS* rs2291738 SNP and the CC genotype in the *TIMELESS* rs7302060 SNP were associated with reduced risk of BC in patients with estrogen-/progesterone-positive BC [63]. Chu et al. observed that the A allele in the *NPAS2* rs2305160 SNP was associated with decreased PC risk compared to the GG genotype [50]. Reduced risk for gastric cancer (GC) was observed in carriers of the T allele in the *PER2* rs934945 SNP and carriers of the C allele in the *RORA* rs339972 SNP in a DM [53].

Circadian gene variants have also been associated with patients’ response to anti-tumor therapy. The results reported by Johnson et al. suggest that the toxicity of breast cancer radiotherapy can be reduced by scheduling patients for therapy based on their *PER3* VNTR number and *NOCT* rs131116075 genotypes [64]. This finding is supported by the research by Webb et al., which showed that the time of treatment can influence the toxicity of radiotherapy in patients with breast carcinoma based on their *CLOCK* rs1801260, *PER3* (VNTR; rs2087947) and *RASD1* rs11545787 genotypes [65].

## 3. Circadian Gene Variants in Cardiovascular and Metabolic Diseases

Circadian gene variants have also been associated with various physiological processes and their disruptions, including obesity, different hormone levels, high sterol levels, blood pressure abnormalities, cardiovascular disease, impaired fasting glucose, and diabetes (Table 2).

Circadian gene variants are not only associated with driving factors of obesity in overall population, but also with various risk factors in obese patients.

The research by Corella et al. showed that the G allele in the *CLOCK* rs4580704 SNP was associated with decreased stroke risk in type 2 diabetes patients based on a DM in the Spanish population [70]. Monteleone et al. observed that overweight/obese patients with the CC genotype in the *CLOCK* rs1801260 SNP had significantly higher values of body mass index (BMI) [79] compared to carriers of the T allele in a Caucasian cohort, while Garaulet et al. observed that carriers of the G allele in the same SNP among obese patients were the least responsive to a weight-loss intervention in a population from southeastern Spain [80]. Among patients with essential hypertension in a Chinese population, carriers of the C allele in the *CLOCK* rs1801260 SNP were more susceptible to insulin resistance and were at greater risk of developing high night-time systolic blood pressure [81]. Research by Torrego-Ellacuría et al. showed that carriers of the A allele in the *CLOCK* rs1801260 SNP in a Caucasian cohort showed a greater degree of obesity and significantly lower weight loss and higher weight regain over time after bariatric surgery, regardless of the pre-surgery patient profile [82]. The same research also showed that the TT genotype in the *CLOCK* rs3749474 SNP was associated with morbid obesity in patients who underwent bariatric surgery [82]. However, it was also observed that circadian gene variants could also affect risk factors strictly in non-overweight subjects, as it was shown in a Japanese cohort that C allele in the *CLOCK* rs1801260 SNP was associated with the prevalence of type 2 diabetes in non-overweight subjects, but not in overweight subjects, after adjusting for potential confounding factors, including age, sex, research area, BMI, smoking habit, alcohol drinking status, leisure time exercise, energy intake and family history of diabetes [71]. Other studies have also shown associations between various *CLOCK* variants and obesity indicators, but they did not remain significant after correcting for multiple testing [83,84].

The GG genotype in *BMAL1* rs7950226 SNP was associated with insulin resistance in patients with essential hypertension in a Chinese population [81]. In the research by Woon et al., no significant association between single *BMAL1* SNPs and type 2 diabetes and hypertension were observed in British individuals of European ancestry [85].

Carriers of the CC genotype in the *CRY1* rs2287161 SNP whose carbohydrate intake percentage in total energy intake was increased displayed significant increase in homeostasis model assessment of insulin resistance (HOMA-IR), fasting insulin and a decrease in quantitative insulin sensitivity check index (QUICKI) in a Mediterranean and an European origin North American population [77]. In obese patients who had undergone two years of diet intervention, the A allele of the *CRY2* rs11605924 SNP was significantly associated with a greater reduction in respiratory quotient (RQ) and a greater increase in resting metabolic rate (RMR) and RMR/kg in a predominantly Caucasian cohort [86]. Kovanen et al. did not find any significant associations between specific *CRY1* and *CRY2* SNPs and the metabolic syndrome components after correction for multiple testing [87].

The C allele in the *PER1* rs2585405 SNP was associated with extreme obesity in a Caucasian cohort in the research by Mariman et al., which is a unique finding, given that the C allele produces a functional protein, while the alternative G allele leads to a missense mutation [88]. In a Spanish population, the G allele in the *PER2* rs2304672 SNP and the TT genotype in the *PER2* rs4663302 SNP were associated with a greater probability of withdrawal from dietary treatment for abdominal obesity [89].

In a Mediterranean subgroup, carriers of A allele in *REV-ERBα* rs2314339 SNP whose total fat intake consisted of ≥55% of monounsaturated fatty acids had significantly lower BMI. These results were not observed in a North American subgroup [78].

Additionally, patients of Croatian origin with myocardial infarction were more susceptible to hypertension, type 2 diabetes and disrupted systolic blood pressure, depending on the variant in rs13124436 and rs6811520 SNPs in *CLOCK* and to type 2 diabetes depending on the variant in rs3789327 SNP in *BMAL1* [75].

## 4. Circadian Gene Variants in Sleep Disorders and Psychiatric Diseases

It has been shown that circadian gene variants can determine the preference for morning or evening activity [90,91,92,93], duration of sleep [83], or sleep quality [94]. Mutations in circadian genes have also been directly linked to circadian rhythm sleep disorders (CRSDs), which can be divided into four main types, including advanced sleep-phase disorder (ASPD), delayed sleep-phase disorder (DSPD), irregular sleep–wake rhythm/free-running sleep (FRT) disorder and non-24 h sleep–wake disorder (N-24) [35] (Table 3).

Contrary to the study by Mishima et al. [96], other studies have found no associations between *CLOCK* 3111T/C variants and diurnal preference [105,106]. However, *CLOCK* 3111T/C SNP has been associated with the recurrence of unfavorable sleep phenotypes in patients diagnosed with different psychiatric disorders. The CC genotype in the *CLOCK* 3111T/C SNP was associated with higher recurrence of initial, middle and early insomnia, with reduced need for sleep in patients diagnosed with bipolar disorder (BP) [107], as well as with a higher recurrence rate of illness (number of illness episodes/duration of illness in years) in BP patients [108]. It was also observed among depressed bipolar patients that carriers of the C allele were more active in the evening and slept less compared TT homozygotes, even though the severity of the depression was similar among all patients [109]. Patients diagnosed with major depressive disorder (MDD) who were homozygotes for the C allele in the *CLOCK* 3111T/C SNP had higher recurrence of initial insomnia compared to carriers of the T allele [107].

Circadian gene variants have also been associated with various psychiatric diseases independently of their effect on the sleeping patterns of patients [36]. Seasonal patterns of (hypo)manic and depressive phases, which are found in about 25% of BP patients, were associated with five SNPs in *NPAS2* in a French Caucasian cohort [110]. *PER3* rs228697 was associated with MDD, independently of the patients’ gender, in a Caucasian cohort. When that cohort was stratified by gender, it was found that *PER3* rs228697 SNP was also associated with MDD in female patients, while *CLOCK* rs1801260 SNP was associated with MDD in male patients [111]. *CLOCK* variants have been found to be associated with a wide array of psychiatric disorders [112], but the clinical significance of these associations still remains undefined.

Among patients with MDD who had been treated with selective serotonin reuptake inhibitor (SSRI), it was found that the *PER3* rs228697 CC genotype was associated with a higher sleep factor score compared with the CG genotype. Heterozygotes (TC) in the *PER3* rs228729 SNP had higher risk of suffering from excitement/agitation, akathisia and weight loss compared to the CC homozygotes. Additionally, patients with the AA genotype and the GA genotype in the *PER3* rs10746473 SNP were more likely to suffer from dizziness and tachycardia, respectively, when compared to patients with the GG genotype [113]. Insomnia was significantly more present in carriers of the C allele in the *CLOCK* 3111T/C SNP during antidepressant treatment with fluvoxamine or paroxetine [114]. These findings suggest the possibility that circadian gene genotyping could be useful for the prediction of adverse effects from psychopharmaceuticals. The study by Gyorik et al. suggested that *CLOCK* variants could play a role in mediating stress-induced circadian deregulation, leading to depression in a Caucasian cohort [115], thus offering a new direction for further research of a very relevant topic in modern times.

## 5. Circadian Gene Variants in Neurodegenerative Diseases

In the research by Bacalini et al., an association was found between *PER1* rs3027178 SNP and Alzheimer’s disease (AD), with the G allele having a protective effect for AD [116]. The C allele carriers in the *CLOCK* 3111T/C SNP in an Italian cohort with a history of blood hypertension had a higher risk of conversion to AD than C allele carriers without hypertension [117].

The CC genotype in *BMAL1* rs3789327 SNP and the CC genotype in the *CLOCK* rs6811520 SNP were found to be associated with higher risk for multiple sclerosis (MS) in a Caucasian cohort of Slavic origin [118], but these results were not replicated in a Spanish cohort [119].

In a Chinese population, *BMAL1* rs900147 and *PER1* rs2253820 SNPs were associated with Parkinson’s disease (PD). Additionally, *BMAL1* rs900147 SNP was significantly associated with the tremor-dominant (TD) subtype of PD, while *PER1* rs2253820 SNP was significantly associated with the postural instability and gait difficulty (PIGD) subtype of PD [120]).

## 6. Circadian Gene Variants in Other Diseases

The role of circadian gene variants has also been explored in some diseases that do not fall under any of the categories described in this review so far.

Mutation in *NPAS2* gene causing Leu/Ser substitution at the 471 position has been suggested to be a risk factor for seasonal affective disorder (SAD), but this result was not confirmed in another group of patients [91].

The C allele in the *PER1* rs2585405 SNP has been shown to be a risk factor for noise-induced hearing loss (NIHL) among Chinese noise-exposed workers [121].

## 7. Circadian Genes and Immune Response

The immune system also displays a circadian pattern of activation and inhibition, which manifests in different numbers of circulating immune cells and magnitudes of immune response throughout the day [122]. Circadian proteins influence both innate [123] and adaptive immunity [124], as well as the immune response to tumors [125]. The deregulation of circadian genes has also been shown to play a role in autoimmune diseases [126,127]. However, to the best of our knowledge, there have been no studies observing the associations between specific circadian gene variants and the regulation of immune response.

## 8. Future Research

A large amount of research on circadian genes is focused on their differential expression between healthy subjects and patients diagnosed with different diseases at both the mRNA and protein levels. However, the possible genetic contribution of circadian genes to various phenotypes cannot be overlooked, especially in sleep-related disorders. The influence of circadian gene variants on therapy efficiency and on the development of adverse side effects is a promising direction for further research.

## 9. Conclusions

Different circadian gene variants have been associated with various types of diseases, with the *CLOCK* rs1801260 (3111T/C) SNP being the most researched locus over a wide array of diseases. While it is expected that SNPs in circadian genes affect sleeping patterns, the significance of associations between circadian gene variants and other types of diseases is still unclear and requires further research. The significance of the associations varies depending on patients’ ethnicity, type of disease, and sleeping patterns, making it difficult to come to a steadfast conclusion about the roles of these SNPs. Some of the variants show the potential to be of clinical significance, for example when determining the timing of therapy in order to increase the therapy’s efficiency and predicting the adverse effects of therapy.

## Figures and Tables

**Table 1:** Tumor types and circadian gene variants associated with increased risk for tumor development.

Tumor Type	Gene	SNP ID	Genotype	Population	Reference
Breast cancer	*PER1*	rs2735611	*AA* RM	Caucasian (Polish)	[43]
	*PER2*	rs934945	*CT + TT* DM	Caucasian (Polish)	[43]
	*PER3*	rs57875989	*4/5 + 5/5 VNTR* DM	Caucasian	[44]
	*CLOCK*	rs3805151	*CT + TT* DM	Chinese	[45]
		rs11133373	*CG + GG* DM	Korean	[46]
	*CRY2*	rs10838524	*AG + GG* DM	Caucasian (Polish)	[43]
	*RORA*	rs1482057		French from two administrative areas	[47]
		rs12914272		French from two administrative areas	[47]
	*NPAS2*	rs2305160	*AG*	predominantly Caucasian	[48]
Prostate cancer	*BMAL1*	rs7950226	*GA + AA* DM	Caucasian	[49]
	*CRY2*	rs1401417	*GC + CC* DM	Chinese	[50]
	*NPAS2*	rs1369481	*GA + AA* DM	Caucasian	[49]
		rs895521	*GA + AA* DM	Caucasian	[49]
		rs17024926	*TC + CC* DM	Caucasian	[49]
	*CSNK1E*	rs1534891	*TT*	Caucasian	[49]
Lung cancer	*PER3*	rs228729	*GT + GG* DM	Brazilian	[51]
	*BMAL1*	rs3816360	*CC*	northeast Chinese	[52]
		rs2290035	*AA*	northeast Chinese	[52]
Gastric cancer	*NPAS2*	rs895520	*AA* RM	Caucasian	[53]
Non-Hodgkin lymphoma/B-cell lymphoma	*CRY2*	rs11038689	*GG* RM	predominantly Caucasian	[54]
		rs7123390	*AA* RM	predominantly Caucasian	[54]
		rs1401417	*CC* RM	predominantly Caucasian	[54]

SNP—single-nucleotide polymorphism; RM—recessive genetic model; DM—dominant genetic model.

**Table 2:** Circadian gene variants associated with various phenotypes originating from disrupted physiological processes.

Gene	Variant	Genotype	Phenotype	Population	Reference
*PER1*	rs2585405	*GC + CC*	Lower 3α-diol, higher SHBG	Chinese	[66]
*PER2*	rs6431590	*AG + AA*	Lack of overnight blood pressure decrease	Chinese	[67]
*PER3*	rs57875989	*4/5 + 5/5 VNTR*	Increased serum IGF-I levels andIGF-I:IGFBP3 ratio	Chinese	[66]
*CLOCK*	rs4580704	*CG + GG* DM	Lower blood pressure, higher serum concentrations of MCP1 and adiponectin, lower type 2 diabetes risk	Caucasian or predominantly Caucasian	[68,69,70]
	rs1801260 (3111T/C)	*GG* RM	Higher fasting insulin, higher HOMA-IR	Caucasian	[68]
		*CT + CC*	Higher oddsratio for the prevalence of diabetes	Japanese	[71]
			Higher risk for cardiovascular disease	multiple	[72]
	rs1554483	*GC + GG*	Overweight, obesity	Caucasian	[73]
	rs4864548	*AG + AA*	Overweight, obesity	Caucasian	[73]
	rs13113518	*CT* AM	Higher campesterol levels	Caucasian	[74]
	rs35115774	*C-* AM	Lower campesterol and sitosterol levels	Caucasian	[74]
	rs6832769	*AG* AM	Lower campesterol and sitosterol levels	Caucasian	[74]
	rs3749474	*TC + TT* DM	Higher energy intake, decreased serum levels of IL-6 and MCP1	predominantly Caucasian	[69]
	rs6811520	*TT* RM	Higher incidence of myocardial infarction	Caucasian (Croatian)	[75]
	rs13124436	*GG* RM	Higher incidence of myocardial infarction	Caucasian (Croatian)	[75]
*BMAL1*	rs6486121	*TC + CC* DM	Higher campesterol levels	Caucasian	[74]
	rs3789327	*AG + GG* DM	Lower incidence of myocardial infarction	Caucasian (Croatian)	[75]
	rs12363415	*AG + GG* DM	Lower incidence of myocardial infarction	Caucasian (Croatian)	[75]
	rs3816358	*TT + TG*	Lack of overnight blood pressure decrease	Chinese	[67]
	rs7950226	*GG* RM	Lower risk for MetS comorbidities	multiple	[76]
*CRY1*	rs2078074	*CC* RM	Higher sitosterol levels	Caucasian	[74]
	rs2287161	*CC*	Higher carbohydrate intake	Spanish and Northamerican (predominatly Caucasian)	[77]
*RORββ*	rs1410225	*TT*	Presence of overnight blood pressure decrease	Chinese	[67]
*RORα*	rs10519096	*AG + AA*	Lack of overnight blood pressure decrease	Chinese	[67]
*REV-ERBα*	rs2314339	*AG + AA*	Lower probability of abdominal obesity, more physical activity	Spanish and Northamerican (predominatly Caucasian)	[78]
*NPAS2*	rs3888170	*CT + CC*	Lack of overnight blood pressure decrease	Chinese	[67]
	rs2305160	*GA + AA*	Decreased levels of free and bioavailable testosterone	Chinese	[66]

3α-diol—5a-androstane-3α, 17β-diol glucuronide; SHBG—sex hormone-binding globulin; IGF-I—insulin-like growth factor I; IGFBP3—insulin-like growth factor binding protein 3; DM—dominant model; RM—recessive model; HOMA-IR—homeostasis model assessment of insulin resistance; AM—additive model; RQ—respiratory quotient; RMR—resting metabolic rate; MetS—metabolic syndrome.

**Table 3:** Circadian gene variants associated with various phenotypes originating from disrupted sleeping patterns.

Disorder	Gene	Mutation	Modification	Phenotype	Population	Reference
(F)DSPD	*PER2*	G−>A	Val1205Met	sleep–wake phase delay, idiopathic hypersomnia	Japanese	[95]
	*PER3*	4 VNTR	/	association with evening preference		[92]
		5 VNTR	/	association with morning preference		[92]
				delayed sleep phase, association with diurnal preference	predominantly Caucasian	[93]
	*CLOCK*	3111T/C	/	evening preference, significantly delayed sleep onset, shorter sleep time and greater daytime sleepiness in CC hmozygotes	Japanese	[96]
	*CRY1*	A−>C	∆ exon 11	enhanced interaction with CLOCK and BMAL1, long-period behavioral and body temperature rhythms with diminished amplitudes		[97]
	*CKIɛ*	G−>A	Ser408Asp	protective effect of A allele against DSPD	Japanese	[98]
(F)ASPD	*PER2*	A−>G	Ser662Gly	advance of sleep, temperature, and melatonin rhythms	A single family with ASPD	[99]
	*PER3*	C−>G	Pro415Ala	habitual early spontaneous awakening	A single family with ASPD	[100]
		A−>G	His417Arg	habitual early spontaneous awakening	A single family with ASPD	[100]
	*CRY2*	G−>A	Ala260Thr	alternation of CRY2 conformation, which results in increase in accessibility and affinity for an E3 ubiquitin ligase FBXL3 and consequently CRY2 degradation	A single family with ASPD	[101]
	*TIMELESS*	C−>T	Arg1081X	destabilization of CRY1/2 and PER1/2 heterodimer, a shortened circadian period or altered entrainment	A single family with ASPD	[102]
	*CKIδ*	A−>G	Thr44Ala	a shorter circadian period	A single family with ASPD	[103]
FRT	*PER3*	C−>G	Pro864Ala	G allele is more common in evening types and in FRT individuals	Japanese	[104]
N-24	*CKIɛ*	G−>A	Ser408Asp	protective effect of A allele against N-24	Japanese	[98]

(F)DSDP—(familial) delayed sleep-phase disorder; (F)ASPD—(familial) advanced sleep-phase disorder; FRT—free-running type; N-24—non-24 h sleep–wake syndrome.
